# The preparation of large surface area lanthanum based perovskite supports for AuPt nanoparticles: tuning the glycerol oxidation reaction pathway by switching the perovskite B site

**DOI:** 10.1039/c5fd00187k

**Published:** 2016-04-13

**Authors:** Christopher D. Evans, Simon A. Kondrat, Paul J. Smith, Troy D. Manning, Peter J. Miedziak, Gemma L. Brett, Robert D. Armstrong, Jonathan K. Bartley, Stuart H. Taylor, Matthew J. Rosseinsky, Graham J. Hutchings

**Affiliations:** a Cardiff Catalysis Institute , School of Chemistry , Cardiff University , Main Building, Park Place , Cardiff , CF10 3AT , UK . Email: kondratsa@cardiff.ac.uk; b Department of Chemistry , University of Liverpool , Crown Street , Liverpool , L69 7ZD , UK

## Abstract

Gold and gold alloys, in the form of supported nanoparticles, have been shown over the last three decades to be highly effective oxidation catalysts. Mixed metal oxide perovskites, with their high structural tolerance, are ideal for investigating how changes in the chemical composition of supports affect the catalysts' properties, while retaining similar surface areas, morphologies and metal co-ordinations. However, a significant disadvantage of using perovskites as supports is their high crystallinity and small surface area. We report the use of a supercritical carbon dioxide anti-solvent precipitation methodology to prepare large surface area lanthanum based perovskites, making the deposition of 1 wt% AuPt nanoparticles feasible. These catalysts were used for the selective oxidation of glycerol. By changing the elemental composition of the perovskite B site, we dramatically altered the reaction pathway between a sequential oxidation route to glyceric or tartronic acid and a dehydration reaction pathway to lactic acid. Selectivity profiles were correlated to reported oxygen adsorption capacities of the perovskite supports and also to changes in the AuPt nanoparticle morphologies. Extended time on line analysis using the best oxidation catalyst (AuPt/LaMnO_3_) produced an exceptionally high tartronic acid yield. LaMnO_3_ produced from alternative preparation methods was found to have lower activities, but gave comparable selectivity profiles to that produced using the supercritical carbon dioxide anti-solvent precipitation methodology.

## Introduction

The oxidation of alcohols provides a route to carboxylic acids, which are components in many chemical syntheses, including those in the fine chemical and pharmaceutical industries. To achieve this transformation in the liquid phase, low pressures, low temperatures and the use of molecular oxygen as the oxidant are industrially and environmentally advantageous. The oxidation of glycerol, in particular, has attracted significant attention due to its high functionality and its availability from the *trans*-esterification of triglycerides, as a by-product of the biodiesel manufacturing process.^1^

Glycerol can be oxidised with heterogeneous catalysts to produce a range of molecules with applications in polymers, building, cosmetics, food additives, and organic syntheses.^2^ Gold nanoparticles have been shown to be active for the oxidation of glycerol in the presence of a base, such as sodium hydroxide.^3,4^ In these cases, the highest selectivity was to the C3 oxidation product, glyceric acid. Following this work, investigations into the precious metal present on the catalyst were undertaken. It was found that a synergistic effect was in operation when gold was alloyed with another metal, such as palladium^5^ or platinum.^6^

**Scheme 1:**
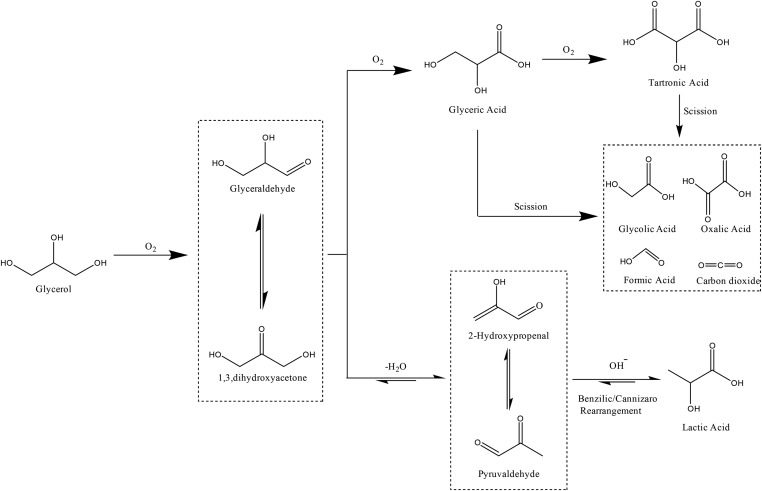
Possible reaction pathways for glycerol oxidation.

The reaction mechanism (shown in Scheme 1) contains multiple steps with a variety of different possible products. The initial step of the oxidation of glycerol is the formation of glyceraldehyde, which is in equilibrium with dihydroxyacetone. In the presence of a catalyst and base, under oxidising conditions, glyceraldehyde has been shown to rapidly oxidise to glyceric acid, which can then be oxidised further.^7^ Recently, further attention has been given to the transformation of glycerol to lactic acid, under oxidative conditions.^8,9^ Lactic acid has many uses in the food industry and also it can be polymerised to poly-lactic acid, a biodegradable material.^10^ This reaction pathway proceeds *via* the dehydration of glyceraldehyde or dihydroxyacetone to form pyruvaldehyde, which then re-arranges into lactic acid.

The effect of a support on the oxidation of glycerol under basic conditions has been studied in detail.^11–13^ Carbon supports have been shown to be more active than titania and iron oxide supports.^12^ A study with Au/NiO and Au/NiO_1–*x*_(TiO_2_)_*x*_ showed a very high activity with the NiO support, but a poor selectivity to any particular product.^14^ Monometallic Au, Pd and Pt supported on activated carbon have been shown to be active for glycerol oxidation under base free conditions.^15^ Further studies have shown that TiO_2_,^16^ MgAl_2_O_4_ and H-mordenite supported gold catalysts have activity for glycerol oxidation under base free conditions. Villa *et al.* studied the effect of the acid and base properties of a support on the activity and selectivity of Au catalysts for the base free oxidation of glycerol.^17^ The study found that basic supports resulted in a high activity, but with the production of a large number of C_1_ and C_2_ scission products, while acid supports had a lower activity but with improved selectivity towards glyceraldehyde.

Evidently, the support structure has a significant impact on the activity and selectivity of Au and Au alloy catalysts for the oxidation of glycerol. Further study, by systematically altering a property of the support, would be desirable. Metal oxide and mixed metal oxide supports offer a huge range of different metal cations to change the properties, such as the acidity/basicity, metal-support interaction or oxygen adsorption capacity. A key issue with respect to such a study is that in many cases changing the metal cation results in a change in the support structure. This, in turn, results in significant variation in the surface area, surface species and morphologies.

Perovskites have the general formula ABO_3_, where cation A is larger than cation B. An interesting aspect of these structures is the fact that the cations, A and B, can be varied, and in so doing, the intrinsic properties of these different perovskites can be tuned to achieve the most desirable characteristics, without affecting the crystal structure of the compound.^18^ This would allow for a systematic study of the effect of different transition metal B sites on the activity and selectivity of a liquid phase oxidation reaction, such as glycerol oxidation. Studies on lanthanum based perovskites for the oxidation of propane and iso-butene have shown that the activity is highly dependent on the choice of B site element (Cr, Mn, Fe, Co or Ni).^19^ Due to the isostructural nature of these LaBO_3_ compounds, correlations could be drawn between the activity and B site electronic configuration. Unfortunately, traditionally prepared perovskite structure materials have been synthesised by precipitation methods, which yield small surface area powders in the range of 1–15 m^2^ g^–1^ (ref. 20 and 21), which, in general, are not ideal for supporting metal nanoparticles.

The preparation of transition metal oxide^22^ and mixed metal oxide catalysts^23^ by a supercritical anti-solvent (SAS) process has been shown to produce large surface area, high activity materials for oxidation reactions. Materials prepared by this method have also been demonstrated to be excellent catalyst supports for precious metal nanoparticles for reactions such as benzyl alcohol oxidation and the direct synthesis of hydrogen peroxide.^24,25^ Utilising this preparation methodology would allow for the preparation of large surface area perovskites. In this work, we investigate the use of perovskite supported precious metal alloy nanoparticles in order to tune the selectivity of the glycerol oxidation reaction. Reaction conditions that facilitate the broadest number of reaction products, including lactic acid, were chosen.

## Experimental

### Perovskite support preparation

**Fig. 1:**
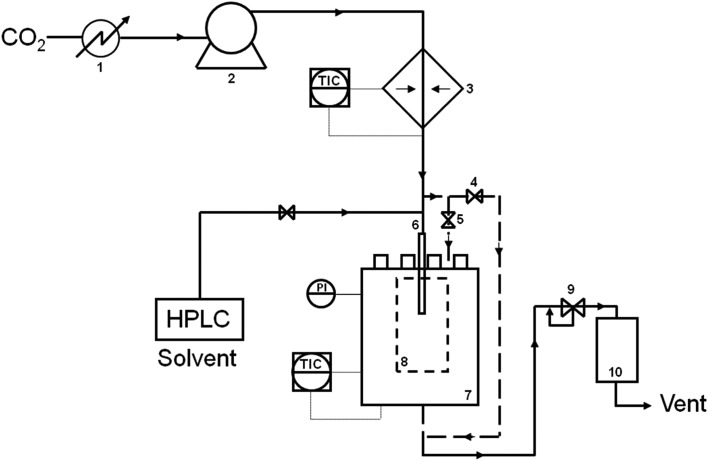
Technical diagram of the Separex SAS equipment. (1) Chiller; (2) liquid pump; (3) heat exchanger; (4) and (5) by-pass valves, (6) co-axial nozzle for CO_2_ and metal salt solution delivery; (7) precipitation vessel; (8) sample recovery vessel; (9) back pressure regulator and (10) separation vessel.

A range of LaBO_3_ (B denotes Cr, Mn, Fe, Co or Ni) perovskites were prepared using the supercritical anti-solvent (SAS) precipitation method. A brief summary of the preparation method is given below, with a more detailed experimental method reported elsewhere.^22^ Lanthanum(iii) acetylacetonate hydrate (4 mg ml^–1^) and one of the B element acetate salts (concentration varied to give the La : B molar ratios shown in Table 1) (Sigma Aldrich ≥99% Puriss) were dissolved in methanol (reagent grade, Fisher Scientific). SAS experiments were performed using apparatus manufactured by Separex. A technical diagram of the SAS apparatus is shown in Fig. 1. CO_2_ (BOC) was pumped through the system (held at 130 bar, 40 °C) *via* the outer part of a co-axial nozzle at a rate of 12 kg h^–1^. The metal salt solution was co-currently pumped through the inner nozzle using an Agilent HPLC pump at a rate of 4 ml min^–1^. The resulting precipitate was recovered on a stainless steel frit, while the CO_2_–solvent mixture passed down stream, where the pressure was decreased to separate the solvent and CO_2_. The precipitation vessel has an internal volume of 1 L. Precipitation was carried out for 120 min followed by a purge of the system with CO_2_ for 30 min under 130 bar and 40 °C. The system was then depressurised and the dry powder collected. The SAS precipitates were then calcined at 750 °C (with a ramp rate of 2 °C min^–1^) for 4 h to produce the perovskite materials.

**Table 1:** Metal salts used for the SAS precipitations and the physical properties of the resultant perovskites

Sample	B metal salt	Precursor solution La : B molar ratio	Precipitated La : B molar ratio^*a*^	Phase composition from XRD	Crystallite size (nm)	Surface area (g m^–2^)
LaCrO_3_	Chromium(iii) acetate^*b*^	1 : 1	1 : 1.06	LaCrO_3_, trace La_2_CrO_6_ ^*c*^	6	52
LaMnO_3_	Manganese(ii) acetate tetrahydrate	1 : 1.2	1 : 1.03	LaMnO_3_ (100%)	18	32
LaFeO_3_	Iron(ii) acetate	1 : 1.4	1 : 0.99	LaFeO_3_ (85%), La_2_O_3_ (9%), Fe_2_O_3_ (6%)	21	26
LaCoO_3_	Cobalt(ii) acetate tetrahydrate	1 : 1.1	1 : 1.05	LaCoO_3_ (90%), Co_3_O_4_ (10%)	22	22
LaNiO_3_	Nickel(ii) acetate tetrahydrate	1 : 1.1	1 : 1.07	LaNiO_3_ (90%), La_2_O_3_ (8%), NiO (2%)	15	36

^*a*^Ratios calculated by MPAES.

^*b*^Chromium acetate prepared in house from potassium chromate and sodium acetate.

^*c*^Phase composition of La_2_CrO_6_ could not be quantified.

In addition to the SAS preparation method, LaMnO_3_ was synthesised by mechanochemical milling of the individual oxides and flame spray pyrolysis. The milling procedure used a planetary ball mill (Retsch PM100). La_2_O_3_ and Mn_2_O_3_ were added to a ZrO_2_ milling vessel with six 15 mm ZrO_2_ balls before being ground at 700 rpm for 16 h. The resulting dry powder was recovered and calcined in static air at 700 °C for 4 h.

Flame pyrolysis was performed using custom built equipment. Aqueous La/B nitrate solutions (0.1 M) were sprayed at a rate of 0.5 ml min^–1^ *via* a Sonozap ultrasonic nebuliser (2.8 W, 130 kHz) into a horizontally aligned propane (0.5 L min^–1^) and oxygen (1.4 L min^–1^) flame (0.082′′ diameter stainless steel nozzle). Gas flows were controlled using mass flow controllers. The resulting powder was collected on a water cooled quartz plate 10 cm from the nozzle tip. A typical collection time was 10 minutes.

### Addition of gold/platinum nanoparticles to the perovskite supports

Aqueous solutions of HAuCl_4_ (Johnson Matthey) and H_2_PtCl_6_ (Johnson Matthey) were prepared at the desired concentrations. Polyvinyl alcohol (PVA, 1 wt% aqueous solution, Aldrich, MW = 10 kDa) was freshly prepared and used as the stabilizer. NaBH_4_ (Sigma Aldrich, 0.1 M aqueous solution) was also freshly prepared and used as the reducing agent. To an aqueous mixture of HAuCl_4_ and H_2_PtCl_6_ of the desired concentration (1 : 1 metal weight ratio, 1 wt% total metal in the final catalyst), the PVA solution was added (PVA/(Au + Pt) (w/w) = 0.65) with vigorous stirring for 2 min. NaBH_4_ was then added rapidly such that the NaBH_4_ : total metal ratio (mol : mol) was 7.5. After 1 h of stirring, the mixture was filtered, washed with distilled water and dried at 120 °C for 16 h.

### Catalyst characterisation

The La : B site elemental ratios of the SAS precipitated perovskites were determined by microwave plasma atomic emission spectroscopy (MP-AES) using an Agilent 4100 instrument. The precipitates were dissolved in aqua regia solutions and the La content was determined using the 394.910 and 398.852 nm emission lines. The emission lines used for the B site elements were as follows: 357.688 and 425.433 nm for Cr, 403.076 and 403.307 nm for Mn, 259.940 and 371.993 nm for Fe, 340.512 and 345.351 nm for Co, and 341.476 and 352.454 nm for Ni. The Au content was determined using the 242.795 and 267.595 nm emission lines and the Pt content was determined from the 265.945 and 270.240 nm emission lines. Powder X-ray diffraction (XRD) was used to determine the phase purity of the prepared perovskites. X-ray diffraction data were collected on a PANalytical X'Pert diffractometer, with Cu K_α1_ radiation, operating at 40 kV and 40 mA. Weight fractions of the phases and crystallite sizes were calculated from relative intensity ratio analysis and the Scherrer equation. Surface area analysis was performed on a Quadrasorb BET. The catalyst was pre-treated under vacuum at 250 °C for 2 h before the surface area was determined by 5 point N_2_ adsorption at –196 °C and the data was analysed using the BET method. TEM was performed using a Jeol 2100 microscope with a LaB_6_ filament operating at 200 kV. Samples were prepared by dispersing the powder catalyst in ethanol and dropping the suspension onto a lacey carbon film over a 300 mesh copper grid. XPS was performed using a Kratos Axis Ultra DLD system with a monochromatic Al K_α_ X-ray source operating at 120 W. Data was collected in the hybrid mode of operation, using a combination of magnetic and electrostatic lenses, at pass energies of 40 and 160 eV for high resolution and survey spectra, respectively. NH_3_-Temperature programmed desorption (TPD) was carried out using a Quantachrome Industries ChemBET TPR/TPD chemisorption analyser, fitted with a thermal conductivity detector (TCD). 100 mg of sample was pre-treated for 1 h at 130 °C (15 °C min^–1^) in He (80 ml min^–1^). Ammonia was adsorbed at room temperature for 20 min to ensure saturation. Physisorbed ammonia was then removed at 100 °C (1 h, 15 °C min^–1^) in He (80 ml min^–1^). Chemisorbed ammonia was subsequently desorbed by heating to 800 °C (15 °C min^–1^) in a flow of He (80 ml min^–1^) with the desorption monitored using a TCD, a current of 180 mV, and an attenuation of 1.

### Glycerol oxidation testing and product analysis

Catalyst testing was performed using a 50 ml Radleys glass reactor. The aqueous glycerol (or glyceric acid) solution (0.3 M, containing NaOH (NaOH/glycerol ratio = 4, mol/mol)) was added into the reactor. The reactor was then heated to 80 °C prior to being purged three times with oxygen. Following this, the desired amount of catalyst (glycerol/metal ratio = 1000, mol/mol) was suspended in the solution and the reactor was heated to 100 °C. The system was then pressurised to 3 bar O_2_ and the reaction mixture was stirred at 900 rpm. After the stated reaction time, the reactor vessel was cooled to room temperature and the reaction mixture was diluted by a factor of 10 before being analysed by HPLC (Agilent 1260 infinity HPLC) equipped with ultraviolet and refractive index detectors and a Metacarb 67H column (held at 50 °C). The eluent was an aqueous solution of H_3_PO_4_ (0.01 M), used at a flow rate of 0.8 ml min^–1^. Quantification of the reactants consumed and products generated was determined by an external calibration method. The reaction effluent was analysed for the following products: glyceric acid, tartronic acid, oxalic acid, glycolic acid, formic acid, acetic acid and lactic acid.

To study the reusability of the catalyst, four sequential reactions were performed with the catalyst washed with water, filtered and oven dried (120 °C, 16 h) between reactions.

## Results

### Properties of the SAS precipitated perovskites

As observed in Table 1, the precipitation of near stoichiometric La and B elements from the SAS precipitations was achieved for all the different perovskites, which is an important factor when producing perovskite materials with high phase purity. However, in some cases, this required an excess of the B site metal salt in the metal salt solution, to prevent excess La in the final precipitate. Non-stoichiometric precipitation was due to the different precipitation yields of the individual metal acetate salts, dictated primarily by the solubility of the salts in supercritical CO_2_–methanol under the conditions used. It is envisaged that the yields from the SAS process could be altered to give precipitate ratios closer to 1 : 1 from initial 1 : 1 starting solutions by varying the pressure, solvent : CO_2_ ratio and also the solution injection geometry.^26^ Given that the purpose of using the SAS technique was to access novel morphologies and large surface areas for catalyst discovery and not catalyst production, the intensive research required to optimise each perovskite preparation was not considered essential and alteration of the initial starting salt ratios was a simpler way of precipitating materials with the correct stoichiometry.

**Fig. 2:**
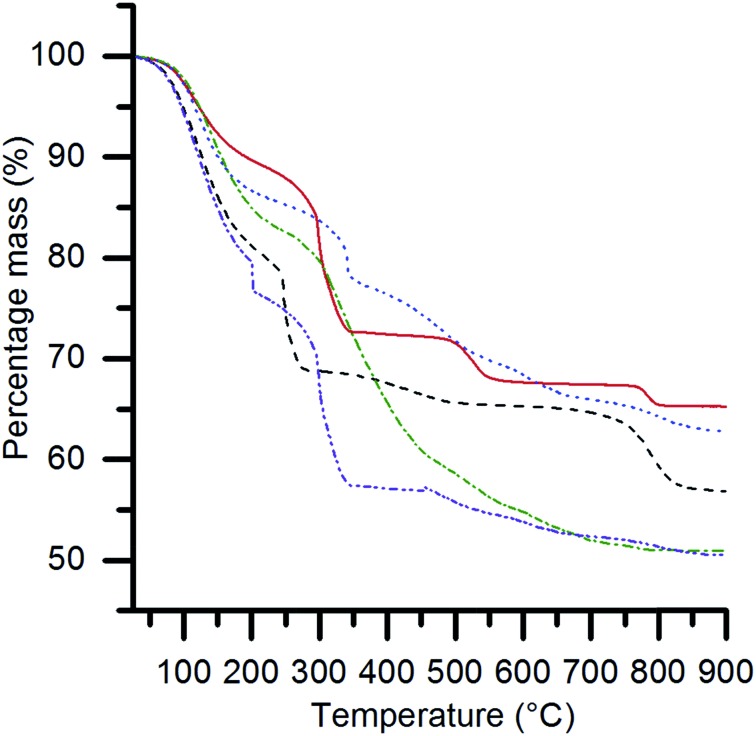
Thermogravimetric analysis of the SAS precipitated materials, where the B site element is: Cr (red solid); Mn (dashed black); Fe (blue dotted); Co (green dashed-dotted); and Ni (purple dashed-dotted). The experimental conditions used were: 10 °C min^–1^ ramp rate, 50 ml min^–1^ flow rate of air and 15–25 mg of sample.

Thermogravimetric analysis (TGA) (Fig. 2) showed multiple stage mass losses for all of the SAS precipitated materials. Previously, we have shown that the SAS precipitation of Ce, Mn, Fe, Co and Ni acetate salts resulted in an acetate salt being retained.^22,25^ However, the local coordination geometry around the metal is altered and the sample no longer displays long range order, according to XRD analysis. This results in their thermal decomposition at temperatures below 400 °C to form their corresponding oxides. It is, therefore, likely that the mass losses observed up to *ca.* 450 °C are indicative of the decomposition of acetate species, with higher temperature mass losses being associated with the transitions between various metal oxide and mixed metal oxide phases. The number of ternary oxide permutations is dependent on the ability of the B site element to adopt different valence states. An example of an extremely complicated system is the La–Cr–O system, where a number of ternary oxide phases are possible (LaCrO_4_, La_2_Cr_3_O_12_, La_2_CrO_6_ and LaCrO_3_) depending on the phase composition and temperature.^27^ These phases decompose at specific temperatures to produce LaCrO_3_ and O_2_. Potentially, the mass losses centred at 520 °C and 785 °C represent phase decomposition of La_2_Cr_3_O_12_ and La_2_CrO_6_, respectively. Detailed *in situ* XRD analysis could provide more information on these intermediate states, although this is not included in the scope of the current work. The SAS precipitated materials were calcined at 750 °C, as TGA showed the final mass loss event had started before this temperature.

**Fig. 3:**
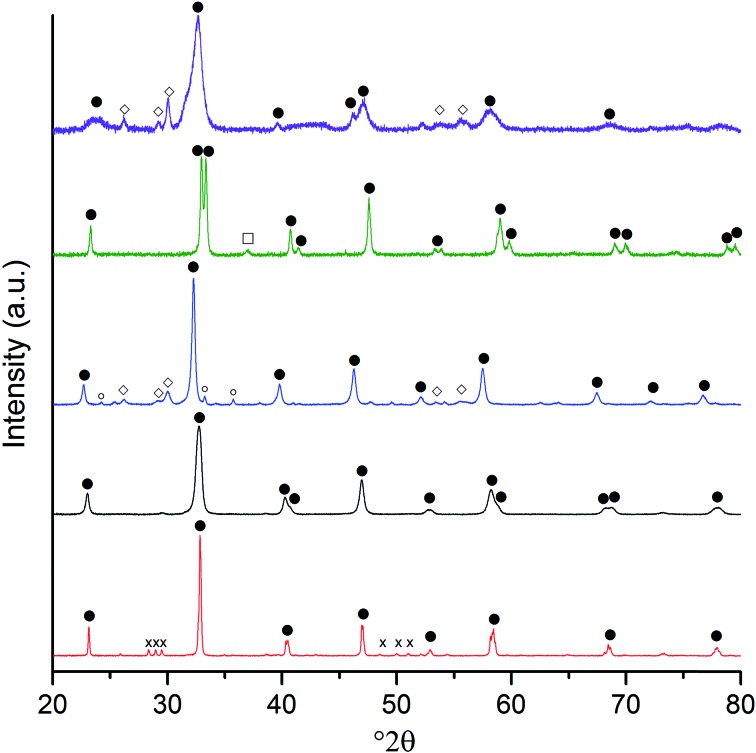
Powder X-ray diffraction patterns of the SAS La : B precipitates after 750 °C calcination. In ascending order, the B site samples are: Cr (red); Mn (black); Fe (blue); Co (green); and Ni (purple). Phases: 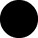 perovskite phases (for simplicity, the rhombohedral, orthorhombic and cubic phases are not differentiated); 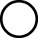 Fe_2_O_3_; 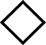 La_2_O_3_; 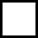 Co_3_O_4_; and 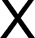 La_2_CrO_6_.

XRD analysis (Fig. 3 and Table 1) of the calcined materials showed that perovskite phases were dominant. Discernible amounts of by-product phases that contributed to 10 to 15 wt% of the sample were observed in the LaFeO_3_, LaCoO_3_ and LaNiO_3_ samples. The by-phases were the single component La and B site oxides, which could be minimised by higher temperature calcination at the expense of the catalyst surface area. Considering the relatively low composition of the by-product phases, it was concluded that the loss of surface area from a higher temperature calcination would be counterproductive. The other by-product phase of note was a small amount of La_2_CrO_6_ in the LaCrO_3_ sample, which could not be quantified by the relative intensity ratio (RIR) method due to the limited data available for this metastable phase. However, the area of the principle reflection at 28.13° 2*θ* was substantially smaller than that of the LaCrO_3_ phase, suggesting that this Cr(vi) phase was only present in trace quantities.

The crystallite sizes of the SAS precipitated perovskites were calculated from the Scherrer equation, and were found to be between 6 and 22 nm (Table 1). Relative to perovskites prepared by more conventional methods, the crystallite sizes observed from the SAS precipitations were relatively small. This can be attributed to the expected small primary particle size of the SAS precipitate and the relatively low calcination temperature used, which was made possible by the high degree of A and B element mixing afforded by the SAS technique. The small particle size of the SAS precipitated perovskites resulted in surface areas in the region of 22–52 m^2^ g^–1^, which are larger than the 1–15 m^2^ g^–1^ areas found for perovskites prepared by more conventional techniques.^20,21^ The combination of a large surface area and small crystallite size was envisaged to provide a suitable number of surface sites for the anchoring of metal nanoparticles.

**Fig. 4:**
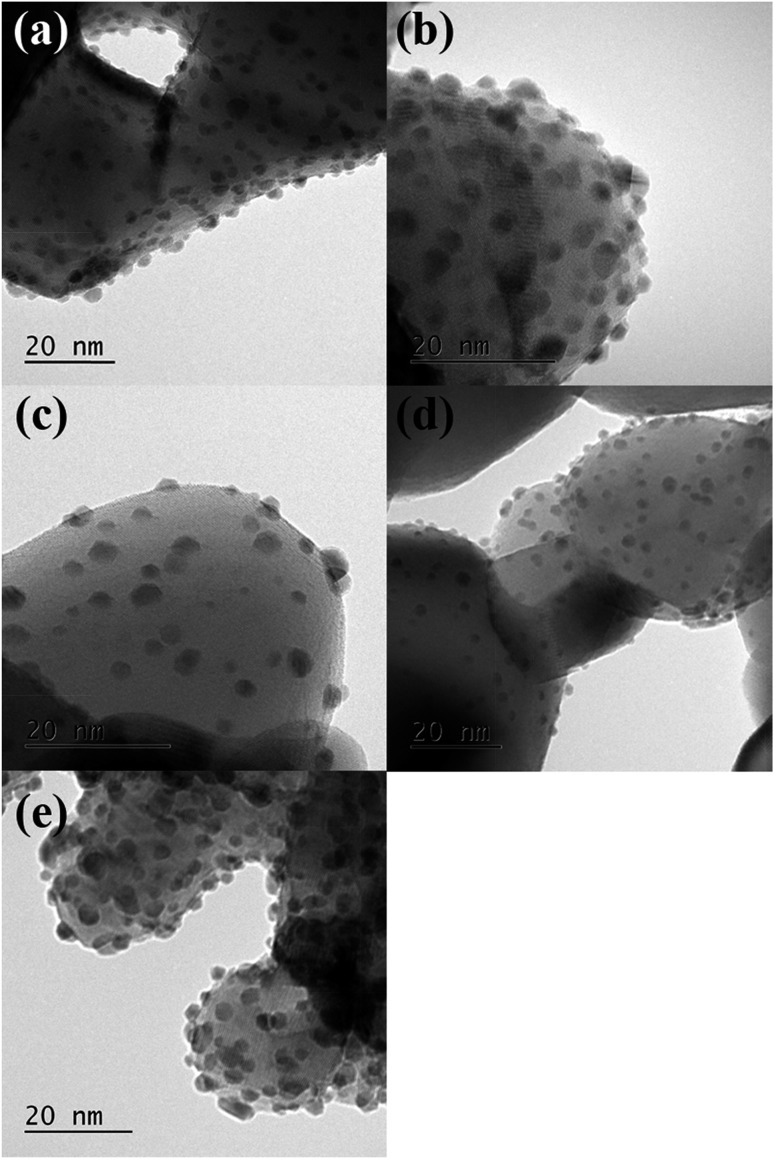
Representative transmission electron micrographs of AuPt supported on the different SAS prepared LaBO_3_ perovskites. (a) AuPt/LaCrO_3_; (b) AuPt/LaMnO_3_; (c) AuPt/LaFeO_3_; (d) AuPt/LaCoO_3_; (e) AuPt/LaNiO_3_.

**Fig. 5:**
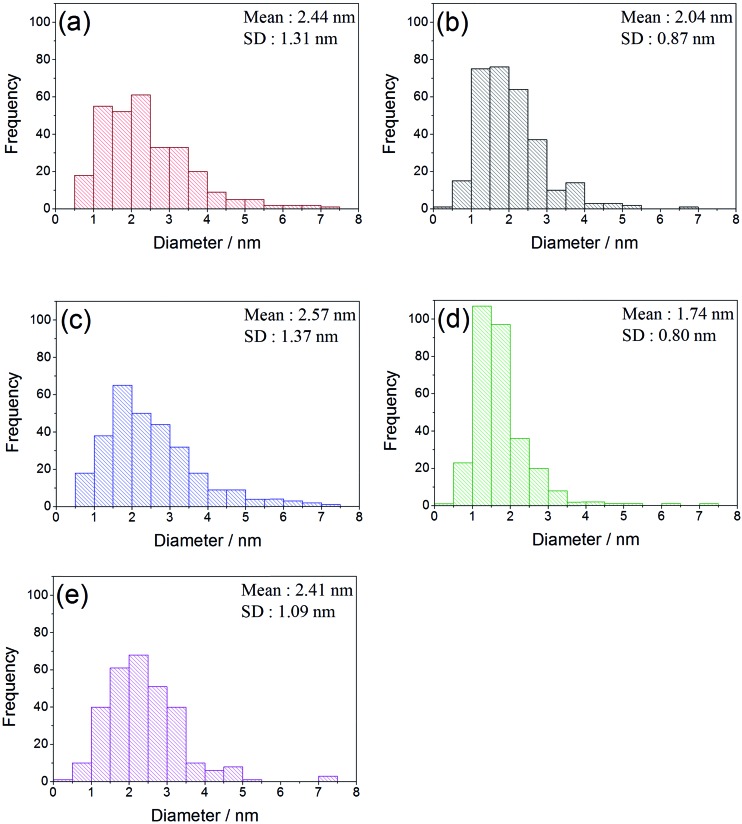
Particle size distribution histograms of AuPt supported on the different SAS prepared LaBO_3_ perovskites. (a) AuPt/LaCrO_3_; (b) AuPt/LaMnO_3_; (c) AuPt/LaFeO_3_; (d) AuPt/LaCoO_3_; (e) AuPt/LaNiO_3_.

The sol immobilisation technique, using PVA as the protecting ligand, was used to deposit 1 wt% AuPt (1 : 1 molar ratio) nanoparticles onto the perovskite materials. For all supports, the desired metal content and Au : Pt ratio was deposited (calculated from MP-AES data shown in Table 2). Representative TEM images, with corresponding particle size distributions, are shown in Fig. 4 and 5. In all cases, a small mean particle size of *ca.* 2 nm with a standard deviation of *ca.* 1 nm was observed. The slight size variation in the metal nanoparticle size between the different perovskite supported catalysts was found to have no strong correlation with either the surface area or the B site element. The observed particle sizes are comparable to those reported for catalysts prepared with more conventionally used supports, such as TiO_2_,^28^ which might be expected as the SAS precipitated perovskites had comparable surface areas to their 50–60 m^2^ g^–1^. The ability of the anti-solvent precipitation methodology to prepare perovskites with sufficient surface areas to successfully support 1 wt% AuPt led us to investigate these materials as catalysts for a liquid phase oxidation reaction.

**Table 2:** Au and Pt surface and bulk composition of the AuPt/perovskite catalysts from MP-AES and XPS analysis

Support	Au and Pt content (wt%)	Bulk Au/Pt ratio from MP-AES	Surface Au/Pt ratio from XPS
LaCrO_3_	0.55 (Au), 0.55 (Pt)	1.0	0.6
LaMnO_3_	0.47 (Au), 0.46 (Pt)	1.0	1.4
LaFeO_3_	0.46 (Au), 0.47 (Pt)	1.0	1.0
LaCoO_3_	0.55 (Au), 0.54 (Pt)	1.0	0.6
LaNiO_3_	0.50 (Au), 0.48 (Pt)	1.1	0.9

**Fig. 6:**
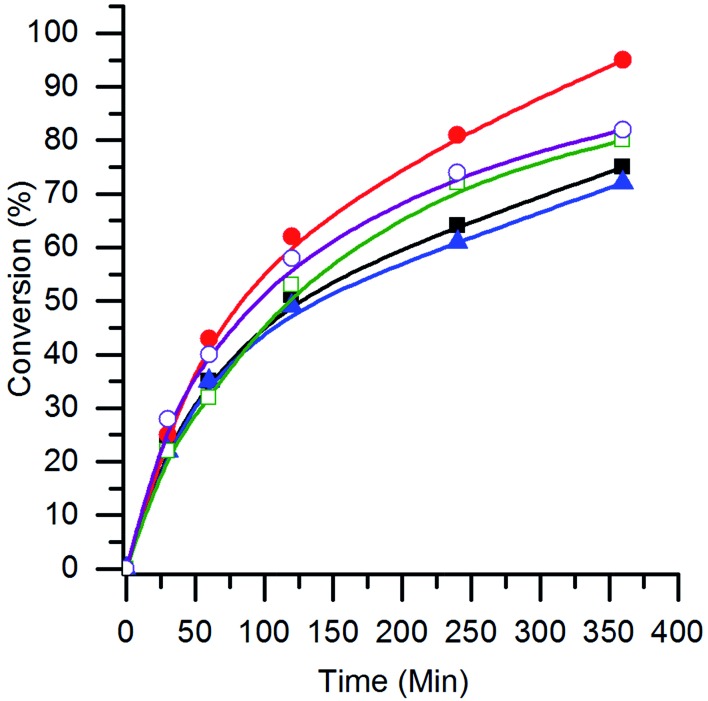
The conversion of glycerol with AuPt/LaBO_3_ catalysts, where the B sites of the supports are: Cr (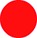); Mn (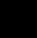); Fe (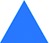); Co (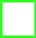); and Ni (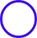). Conditions: glycerol 0.3 M in water, 4 : 1 NaOH : glycerol, glycerol : metal = 1000, 3 bar O_2_, temperature = 100 °C.

The effect of the B site in the AuPt/perovskite catalysts for the glycerol oxidation reaction was investigated. The conversion profiles are shown in Fig. 6 and the turnover frequencies (TOFs) (mol_glycerol converted_ mol_AuPt_^–1^ h^–1^) are given in Table 3. It can be seen that all the catalysts had similar initial rates, with the TOF of the AuPt/LaCrO_3_ and AuPt/LaNiO_3_ catalysts being slightly higher at 620 h^–1^ and 560 h^–1^, respectively, compared with the other catalysts, which had TOFs of 440–460 h^–1^. No correlation between the TOF and the AuPt nanoparticle size was observed, although this was expected as the variance in TOFs and particle sizes was small. The TOFs observed for the AuPt/perovskite catalysts were found to be comparable to the *ca.* 500 h^–1^ observed by Shen *et al.* when using a 1 wt% AuPt/TiO_2_ catalyst under similar reaction conditions (90 °C with a base : substrate ratio of 4 : 1).^9^

**Table 3:** Glycerol oxidation using AuPt supported on the perovskite and single oxide materials^*a*^

Catalyst	TOF^*b*^ (h^–1^)	Selectivity^*c*^ (%)			
		Glyc^*d*^	Tar^*d*^	C–C scission^*d*^	Lac^*d*^
**Perovskites**					
AuPt/LaCrO_3_	620	5	7	2	86
AuPt/LaMnO_3_	460	70	17	8	5
AuPt/LaFeO_3_	440	10	2	19	69
AuPt/LaCoO_3_	440	43	9	24	24
AuPt/LaNiO_3_	560	30	3	26	41

**Corresponding single oxides**					
AuPt/MnO_2_	560	33	3	33	31
AuPt/Fe_2_O_3_	240	19	2	58	21
AuPt/Co_3_O_4_	180	23	4	24	49
AuPt/NiO	700	30	10	39	21

^*a*^Reaction conditions: glycerol 0.3 M in water, 4 : 1 NaOH : glycerol, glycerol : metal = 1000, 3 bar O_2_, 100 °C.

^*b*^TOF calculated at 30 min, moles of glycerol converted/moles of metal per h.

^*c*^Selectivity calculated after 6 h of reaction.

^*d*^Key: Glyc = glyceric acid, Tar = tartronic acid, C–C scission = C_1_ and C_2_ products, and Lac = lactic acid.

**Fig. 7:**
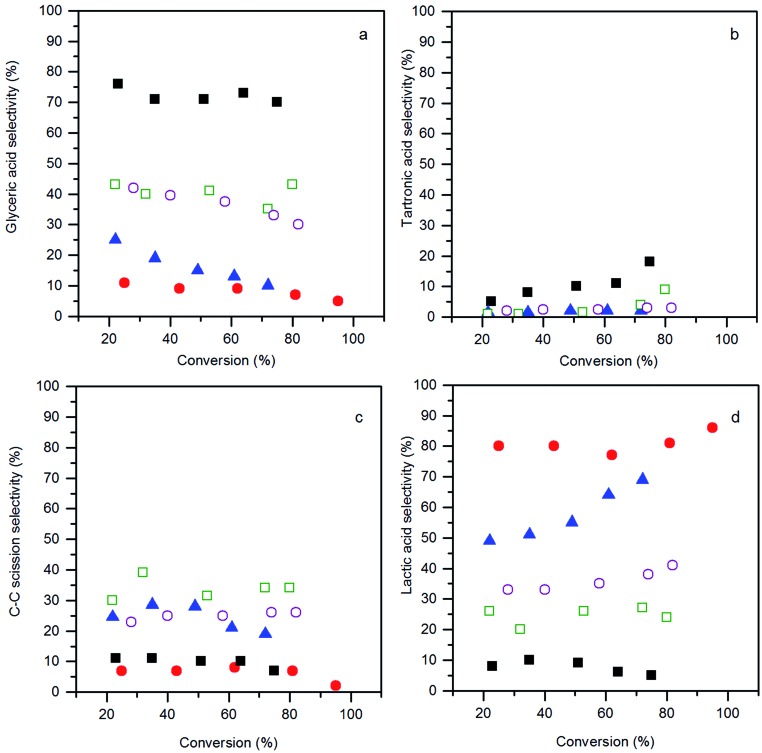
Conversion–selectivity plots: (a) glyceric acid selectivity; (b) tartronic acid selectivity; (c) C–C scission selectivity; and (d) lactic acid selectivity, from the glycerol oxidation reaction using the AuPt/LaBO_3_ catalysts, where the B sites of the supports are: Cr (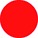); Mn (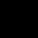); Fe (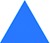); Co (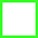); and Ni (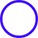). Conditions: glycerol 0.3 M in water, 4 : 1 NaOH : glycerol, glycerol : metal = 1000, 3 bar O_2_, temperature = 100 °C.

The product selectivities with different AuPt/perovskite catalysts are shown in Fig. 7. The activities of the different catalysts were very similar, whereas, the product distributions were markedly different. The LaMnO_3_ supported catalyst was found to favour C_3_ oxidation products, with a high selectivity towards glyceric acid, which at high conversions further oxidised to tartronic acid. Selectivities to C–C scission products (oxalic acid, glycolic acid, formic acid and CO_2_) and lactic acid were low and consistent across the range of conversions observed for the LaMnO_3_ supported catalyst. This is an interesting result as the reaction conditions used are reported to enhance the dehydration and re-arrangement of glyceraldehyde to lactic acid. Under these relatively high temperatures and high base concentrations, AuPt nanoparticles supported on CeO_2_ or TiO_2_ have been reported to give lactic acid selectivities between 60 and 80%.^8,9^ Under milder conditions (for example temperatures of *ca.* 60 °C and a base : glycerol ratio of 2 : 1), where the rate of the lactic acid pathway is subdued, the selectivity profile seen for the AuPt/LaMnO_3_ catalyst is frequently observed.^29^ It is apparent that employing the LaMnO_3_ support switches off the lactic acid pathway and promotes the oxidation pathway.

The selectivity profiles for the LaCoO_3_ and LaNiO_3_ supported catalysts are similar, with a moderate selectivity towards glyceric acid, a relatively high C–C scission selectivity and a lactic acid selectivity of *ca.* 30%. It was noted that for the AuPt/LaNiO_3_ catalyst, the glyceric acid selectivity decreased when the glycerol conversion increased from 28% to 82%. This decrease in the glyceric acid selectivity did not correspond to a further oxidation to tartronic acid, but was accompanied by an increase in lactic acid formation, indicating a change in the prevalence of the oxidation and dehydration reaction pathways.

The selectivity profile of AuPt/LaFeO_3_ also changed with respect to glycerol conversion. At low conversions, the AuPt/LaFeO_3_ catalyst had a glyceric acid selectivity of 25%, a scission product selectivity of 24.5% and 49% selectivity towards lactic acid. As the reaction progressed and the glycerol conversion increased, the selectivity towards glyceric acid decreased dramatically. As observed with the AuPt/LaNiO_3_ catalyst, the change in the selectivity profile was the result of a shift towards lactic acid production, with the selectivity to this product increasing from 49% to 69% over the reaction period. At 69% lactic acid selectivity, the AuPt/LaFeO_3_ catalyst is similar to previously reported catalysts, under comparable conditions.^8^ The shift towards lactic acid formation was far more significant in the LaFeO_3_ supported catalyst than any of the other perovskite supported catalysts. This may suggest that the oxidation sites on this catalyst are not stable or are blocked by reaction intermediates.

The highest lactic acid yield was observed with the AuPt/LaCrO_3_ catalyst, with 86% selectivity towards this product at 95% glycerol conversion. Unlike the LaFeO_3_ supported catalyst, the selectivity towards lactic acid was relatively insensitive to conversion, with only a slight increase in the lactic acid selectivity from 80% to 86% over the full conversion range. With a TOF of 620 h^–1^ and a selectivity towards lactic acid above 85%, the AuPt/LaCrO_3_ catalyst is a highly effective catalyst for lactic acid production from glycerol.

Evidently, the variation of the perovskite B site had a dramatic effect on the glycerol oxidation product distribution. The choice of Mn for the B site resulted in suppression of the lactic acid pathway with glyceric acid being the dominant product. With Cr or Fe B sites, the dehydration pathway to lactic acid is promoted, and a Ni or Co B site produces both oxidation and dehydration products. Both the lactic acid pathway and oxidation pathway to glyceric or tartronic acid proceed *via* the glyceraldehyde intermediate. As the rate limiting step for both reaction pathways is the initial proton abstraction from glycerol to form the alkoxy intermediate, substantially different product distributions were observed alongside very similar catalyst activities.

**Fig. 8:**
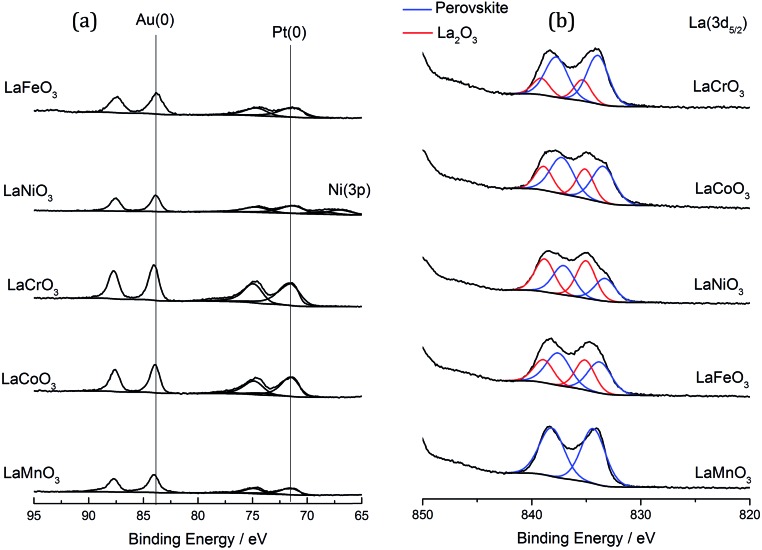
XPS analysis of the AuPt/LaBO_3_ catalysts. (a) Au (4f) and Pt (4f) spectra of the catalysts. (b) La (3d_5/2_) spectra.

The most obvious reason for this variation in activity was that the different perovskite supports altered the metal support interaction of the AuPt nanoparticles. This could result in different nanoparticle morphologies, such as phase separated Au and Pt, random alloy AuPt or core shell morphologies. Given the poor Z contrast between Au and Pt, stands for scanning transmission electron microscopy (STEM) and X-ray energy dispersive spectroscopy (XEDS) analysis of the AuPt nanoparticle morphology is challenging. Modern XEDS detectors, with greatly improved X-ray collection efficiencies, could allow for element mapping of AuPt nanoparticles at low enough energies to limit beam damage and present an opportunity for further development of the current work. In an attempt to gain some initial understanding of the environment and structure of the metal nanoparticles, a combination of conventional TEM, reported earlier in Fig. 4, to investigate the support–particle size dependency, and XPS, to probe the metal oxidation state and relative surface composition, was performed. As mentioned previously, no strong variance in the metal nanoparticle size was observed by TEM, discounting the most obvious effect of metal dispersion on product selectivity. XPS analysis of the Au and Pt 4f levels (Fig. 8 and Table 2) showed that both the Au and Pt were in the metallic oxidation state. As seen in Table 2, the surface ratio of the two metals was noted to vary with the different B sites with a minimum Au : Pt ratio of 0.6 for the Cr and Co containing perovskites and a maximum of 1.4 for the Mn perovskite. Given that the bulk Au : Pt ratios, as determined by MP-AES, were found to be unity for all of the catalysts, the changes in the surface ratio are indicative of changes in the metal nanoparticle structure. This could potentially be due to changes in the composition of the alloy nanoparticles, the presence of monometallic phases or the formation of core–shell structures. Previous studies in which both core–shell and random alloy AuPt nanoparticles were prepared by sol immobilisation showed that the morphologies were well represented by the observed XPS metal ratios (*i.e.* a Au : Pt ratio of 1 : 1 for the random alloy or 0.75 : 1 for the core–shell).^30^ The exact nature of the nanoparticle structure could be investigated further by STEM-XEDS analysis, although this would be challenging.

Under milder reaction conditions, at lower base concentrations and temperatures, where the oxidation pathway is prevalent, monometallic Au catalysts have been found to promote the selective oxidation of glycerol to glyceric acid, while Pt and Pd have been shown to promote over-oxidation to form C–C scission products.^29^ The AuPt/LaMnO_3_ catalyst with the Au surface enrichment was found to be the most selective towards glyceric acid production, while the Pt enriched surface of the AuPt/LaCrO_3_ catalyst selectively produced lactic acid. No strong trend between the Pt surface concentration and C–C scission products was observed. The hypothesis that the Au surface concentration correlates with the promotion of the oxidation pathway does, however, appear to be contradicted by literature reports of the glycerol to lactic acid reaction with supported AuPt catalysts with Au : Pt ratios between 3 : 1 and 1 : 3, which showed that these catalysts had very little variation in activity or selectivity towards lactic acid. Monometallic Au and Pt catalysts did show some slight variation in lactic acid yields (Au = 46% lactic acid yield and Pt = 56% lactic acid yield),^8^ although this difference is far less significant compared to the difference between the AuPt/LaMnO_3_ and AuPt/LaCrO_3_ catalysts (AuPt/LaMnO_3_ = 4% lactic acid yield and AuPt/LaCrO_3_ = 82% lactic acid yield).

Consideration of the properties of the perovskite supports and their potential to alter the reaction pathway must also be considered. XPS analysis (Fig. 8b) showed a discernible surface contribution from La species with an alternative binding energy to that of the perovskite that could be assigned to La_2_O_3_ or La(OH)_3_. It was found that the materials produced with the Fe, Co and Ni B sites had a higher contribution of this phase, which correlates with the increased La_2_O_3_ from XRD analysis. A loose correlation between the increased selectivity towards C–C scission and the content of phase separated La_2_O_3_ and B site oxide was observed, although no correlation with lactic acid or glyceric acid production can be found.

**Fig. 9:**
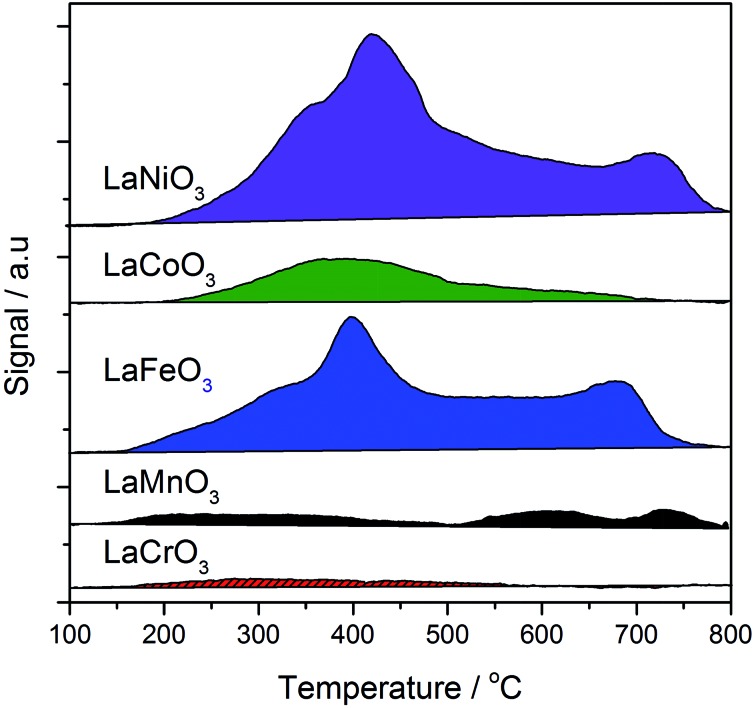
Temperature programmed desorption of ammonia analysis of the SAS prepared LaBO_3_ supports, where B is: Cr (red solid); Mn (black); Fe (blue); Co (green); or Ni (purple).

The total acidity of the perovskites was measured by NH_3_-TPD (Fig. 9). Previous TPD-MS studies have shown that a portion of the adsorbed ammonia is oxidised to nitrous oxide and water using lattice oxygen from perovskites. As such, desorption profiles for perovskites are complex, with four distinct desorbed species: (i) NH_4_^+^ weakly bound to surface hydroxyl groups, (ii) NH_3_ chemisorbed to Lewis acid sites, (iii) N_2_O, formed through oxidation of NH_3_ with lattice oxygen (also yielding H_2_O), and (iv) lattice oxygen. Coordinatively unsaturated metal cations at the surface of single metal oxides act as Lewis acid sites, giving rise to type (ii) and (iii) desorptions, which occur within a temperature range of 150–600 °C. Loss of lattice oxygen as type (iv) desorptions occurs at temperatures in excess of 500 °C. Acid site analysis showed significant desorptions for the Fe, Co and Ni B site samples within the temperature region associated with Lewis acid sites. This is consistent with the XRD data from Table 1, where 10–15% single oxide phase contamination was observed. Within this 10–15%, LaNiO_3_ and LaFeO_3_ were shown to contain mainly trivalent M^*n*+^, whilst LaCoO_3_ contained Co_3_O_4_, which exists in a normal spinel phase as Co^II^Co^III^_2_O_3_. Very little ammonia adsorption was observed for the LaCrO_3_ and LaMnO_3_ samples, which, according to XRD data, contained little single metal oxide phases. It should be noted, however, that in contrast to the XRD data, the XPS data in Fig. 8 shows the presence of a La_2_O_3_ phase, itself a Lewis acid, in all but the LaMnO_3_ perovskite. In this way, the La_2_O_3_ : perovskite ratio followed the order: LaNiO_3_ > LaFeO_3_ > LaCoO_3_ > LaCrO_3_. LaNiO_3_, LaFeO_3_ and, to a lesser extent, LaMnO_3_ exhibited a high temperature desorption at *ca.* 650–700 °C, which might be attributed to the loss of highly mobile lattice oxygen. These data suggest that the more Lewis acidic single oxide phases are responsible for the loss of controlled selectivity in the AuPt/LaFeO_3_, AuPt/LaCoO_3_ and AuPt/LaNiO_3_ catalysts and the formation of C–C scission products.

To test the hypothesis that the single metal oxide phases were responsible for the differences in product selectivity, AuPt was deposited on MnO_2_, Fe_2_O_3_, Co_3_O_4_ and NiO supports prepared by the SAS process (calcined at 750 °C) and tested as catalysts for the glycerol oxidation reaction (Table 3). Unlike the corresponding perovskite supported catalysts, the single oxide supported catalysts had a significant range of TOFs from 180 to 700 h^–1^, with the TOF for the Ni and Mn single oxides being higher than their corresponding perovskite catalysts. It is important to note that all of the catalysts were less selective than the AuPt/perovskites, with the highest selectivity towards any product being 49% lactic acid with the AuPt/Co_3_O_4_ catalyst. Specifically, the high selectivity towards glyceric acid with the AuPt/LaMnO_3_ catalyst (69%) was not replicated with the AuPt/MnO_2_ catalyst (33% glyceric acid selectivity) and the high lactic acid selectivity with the AuPt/LaFeO_3_ catalyst was not replicated compared to the AuPt/Fe_2_O_3_ catalyst (69% *vs.* 21%). As a point of comparison, Villa *et al.* studied Au/NiO and Au/NiO–TiO_2_ (NiO impregnated onto TiO_2_) for glycerol oxidation. Similar to the current study, the NiO supported catalyst was found to be highly active but with a broad range of products. Dilution of the NiO sites by supporting onto TiO_2_ resulted in a significant decrease in the activity and an improved selectivity towards glyceric acid.^14^

**Fig. 10:**
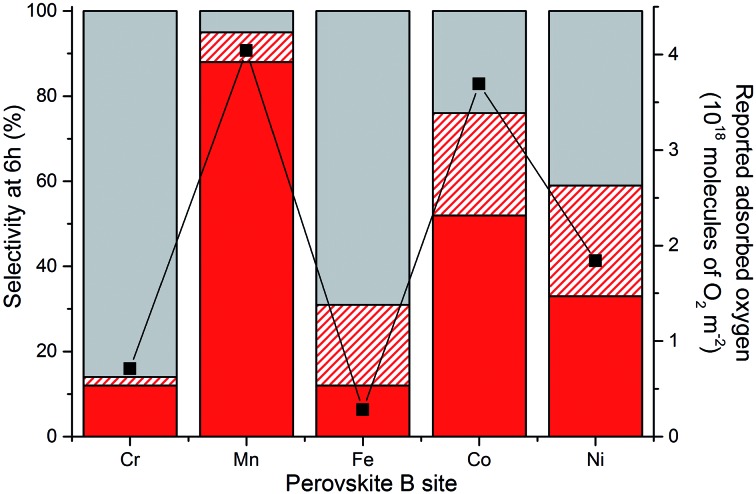
Selectivity profiles of the AuPt/LaBO_3_ catalysts compared to reported oxygen adsorption values for the relevant perovskite phases.^19^ Sum of the C_3_ oxidation products (glyceric and tartronic acid) shown in solid red. C–C scission products (sum of glycolic acid, oxalic acid, formic acid and CO_2_) shown in dashed red. Lactic acid selectivity shown by the solid grey bar. (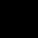) read from the second *y* axis represents the reported oxygen adsorption of the perovskite.

Perovskites have been extensively researched as catalysts for the deep oxidation of alkanes, alkenes and CO.^19,31^ A strong correlation between the activity and O_2_ coverage profiles has been reported, with perovskites with good O_2_ adsorption capacities being more active. Tejuca and co-workers investigated the chemisorption of O_2_ and isobutene on a range of LaBO_3_ catalysts with the same range of B sites used in this study (*i.e.* Cr, Mn, Fe, Co and Ni).^19^ The adsorption profiles of O_2_ on the LaBO_3_ clean surfaces from this earlier study have been plotted against the glycerol oxidation selectivity profiles of the various B sites (at 6 h reaction time) used in AuPt/LaBO_3_, as shown in Fig. 10. If C–C scission products are assumed to be produced from an oxidation process, the sum of the oxidation pathway products correlates well with the reported oxygen adsorption capacities. The most selective catalyst towards the oxidation pathway used the LaMnO_3_ support, which had the best oxygen adsorption capacity. The LaCrO_3_ and LaFeO_3_ supports with poor oxygen adsorption characteristics were found to give catalysts that favour the production of lactic acid, which is formed from an initial oxidation followed by dehydration to pyruvaldehyde and rearrangement. The LaCoO_3_ and LaNiO_3_ supported catalysts were found to produce both oxidation and dehydration products that correspond to the intermediate oxygen adsorption capacities. An interesting point highlighted in Tejuca and co-workers’ paper was that the O_2_ capacities were enhanced on the addition of the iso-butane substrate for all B site perovskites, but disproportionately across the period (*i.e.* the effect was more significant for Co than Mn B sites).^19^ Further studies with more ideal surfaces could probe the effect of glycerol adsorption on influencing oxygen adsorption.

A question raised from observing this range of product distributions when choosing different perovskite supports is to ask whether the support itself is active. Glycerol oxidation testing with the perovskite supports gave conversions of less than 1% after 30 min time on line, demonstrating that the metal oxide system had a very low activity for alcohol oxidation under these conditions. Isotopic labelling and computational studies by Davis and co-workers demonstrated that, on supported Au and Pt catalysts, the role of O_2_ in glycerol oxidation was indirect, with oxygen regenerating the active surface hydroxyl species instead of being directly incorporated into the acid products.^32^ Given the reaction temperature, it is unsurprising that the perovskites themselves were not active. A possible hypothesis is that the ability of the support to adsorb oxygen would affect the ability of the active site to regenerate. Catalysts with poor oxygen adsorption capacities perform the initial oxidation of glycerol to glyceraldehyde but the rate of the second oxidation process to glyceric acid is slower than that of the dehydration pathway to produce lactic acid. The LaMnO_3_ support with its higher oxygen adsorption capacity facilitates the regeneration of the oxidation site, allowing for glyceraldehyde oxidation to glyceric acid. Alternatively, the change in B site electronic configuration from Cr (d^3^) to Ni (d^7^), which is reflected in each LaBO_3_ perovskite’s oxygen adsorption capability, could affect the prevalence of surface species such as glycerol, reaction intermediates or hydroxyl species.

**Fig. 11:**
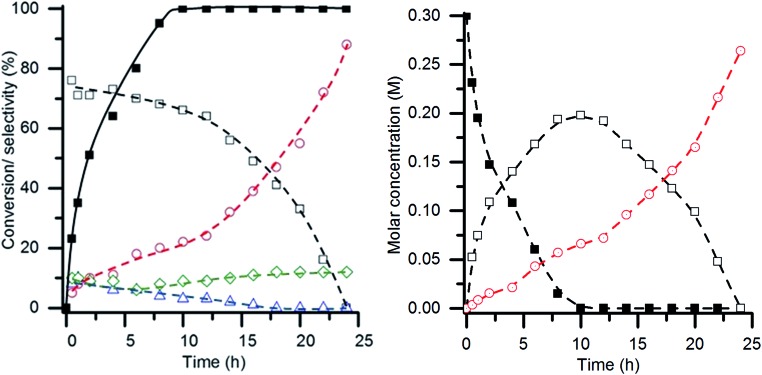
Extended glycerol oxidation reaction time using the AuPt/LaMnO_3_ catalyst. (Left) Time on line conversion and selectivity (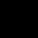 glycerol conversion, selectivity towards: 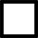 glyceric acid, 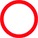 tartronic acid, 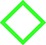 C–C scission, and 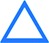 lactic acid). (Right) Time on line molar concentration plot (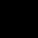 glycerol, 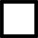 glyceric acid and 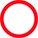 tartronic acid). Conditions: glycerol 0.3 M in water, 4 : 1 NaOH : glycerol, glycerol : metal = 1000, 3 bar O_2_, temperature = 100 °C.

One applied observation of the AuPt/LaMnO_3_ catalyst's ability to promote the selective oxidation pathway under such aggressive conditions (high temperature and base : substrate ratio) was that this catalyst was the only one tested to produce tartronic acid. Given the functionalization of this molecule, it is acknowledged as being a high value added product and would be a desirable molecule to make in high yield. While tartronic acid was observed at low conversions, the selectivity towards it was noted to increase at higher conversions. Given that the glycerol conversion did not reach 100% over the 6 h reaction time using the AuPt/LaMnO_3_ catalyst, an experiment with the reaction time extended to 24 h was performed. The conversion, selectivity profile and molar concentrations with respect to reaction time are shown in Fig. 11. 100% conversion was observed after 10 h time on line, at which point the selectivity towards glyceric acid was 66% with a tartronic acid selectivity of 22%. This represents a slight increase in the tartronic acid selectivity from the 18% observed at 6 h reaction time. After all the glycerol had been converted, it can be seen from the molar concentration plot that glyceric acid is being converted to tartronic acid. Under these reaction conditions, it was clear that the sequential oxidation of glycerol to tartronic acid, *via* glyceric acid, takes place. After 24 h reaction time, all of the glyceric acid had been converted to give a final selectivity towards tartronic acid of 88%, with C–C scission accounting for the remaining products. A yield of 88% tartronic acid in the context of the general literature is exceptionally high and can be attributed to the high base : substrate ratio (4 : 1) and temperature (100 °C) combined with suppression of the dehydration route to lactic acid and excessive C–C scission over the AuPt/LaMnO_3_ catalyst.

**Fig. 12:**
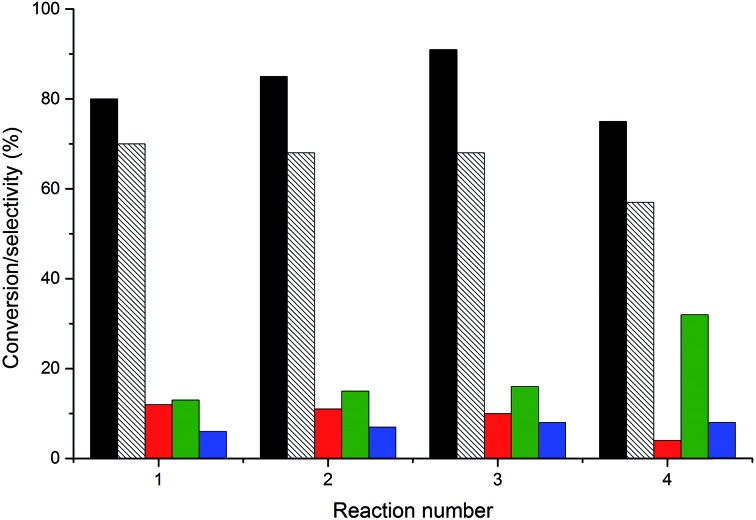
Re-use testing of the AuPt/LaMnO_3_ catalyst. Black fill = glycerol conversion; black dashed = glyceric acid selectivity, red fill = tartronic acid selectivity; green fill = C–C scission selectivity and blue fill = lactic acid selectivity. Conditions: glycerol 0.3 M in water, 4 : 1 NaOH : glycerol, glycerol : metal = 1000, 3 bar O_2_, temperature = 100 °C.

An important consideration in the application of heterogeneous catalysts is their reusability and resistance to leaching. Effluent analysis from reactions using AuPt/LaMnO_3_ and AuPt/LaFeO_3_ by MP-AES is shown in Table 4. These two catalysts were chosen for their different selectivity profiles and also, in the case of the LaFeO_3_ sample, due to the notable changes in selectivity during the reaction. With respect to the B site leaching, less than 2% of the possible metal was found in either reaction effluent. This level of leaching would not contribute to the reaction, as shown by the absence of activity for the perovskite catalysts tested without AuPt. Slightly higher levels of La leaching were determined (between 2 and 6% of the total La), although, again, this had little effect on the reaction. The re-use of the AuPt/LaMnO_3_ catalyst was tested over multiple 6 h reactions, with the conversions and selectivity breakdown for each re-use shown in Fig. 12. Glycerol conversion was noted to increase over the 1st and 2nd re-use tests with the selectivity towards glyceric acid and tartronic acid remaining constant. This slight increase in conversion can be attributed to the removal of the PVA protecting agent under reaction conditions, exposing more active metal surface area. However, the 3rd re-use reaction showed evidence of deactivation with a decrease in conversion and a change in selectivities towards the C–C scission products. Deactivation mechanisms reported for glycerol oxidation include product inhibition from the formation of ketonic species over the surface of the catalysts^33^ or leaching. It was found that no Au and Pt metals had leached from the perovskite supports into the reaction effluent. Due to the minimal leaching observed, product inhibition could be a possible reason for the observed deactivation.

**Table 4:** Effluent analysis following the glycerol oxidation reaction^*a*^

Catalyst	La in effluent (ppm)	B element in effluent (ppm)	% La leaching	% B site leaching
AuPt/LaMnO_3_	58	20	2.1	1.1
AuPt/LaFeO_3_	168	36	6.0	1.9

^*a*^Reaction conditions: glycerol 0.3 M in water, 4 : 1 NaOH : glycerol, glycerol : metal = 1000, 3 bar O_2_, 100 °C, 6 h.

Given the interesting findings from this study of utilising SAS precipitated perovskites as supports for liquid phase oxidation, the viability of perovskites prepared by other routes should be investigated. It is well known that the precursor salts, residual impurities and preparation technique can influence the properties of metal oxide systems. Therefore, the LaMnO_3_ support was prepared by two alternative routes, a mechanochemical synthesis from the single metal oxides and flame pyrolysis of the metal nitrate solutions. It was considered that the mechanochemical preparation route would result in minimal impurities with no precipitation agent or organic species that could leave residues. Flame pyrolysis was anticipated to produce highly crystalline materials with a large geometric surface area and, with further development, could produce materials with comparable surface areas to the SAS precipitation method. The surface areas and crystallite sizes of the prepared LaMnO_3_ supports are shown in Table 5. The small surface area and high crystallite size of the LaMnO_3_ sample prepared by the milling of the single oxides are typical of perovskite materials. The surface area of the material produced by flame pyrolysis was found to be larger than that produced by the mechanochemical preparation route, at 15 m^2^ g^–1^, but not as large as the 32 m^2^ g^–1^ observed for the SAS prepared sample. It should be noted that both the mechanochemical and flame pyrolysis routes were not optimised and that significant work could be undertaken to improve the surface areas and reduce the crystallite sizes.

**Table 5:** Comparison of the physical properties of LaMnO_3_ prepared by different synthetic routes

Preparation method	LaMnO_3_ phase purity^*a*^ (%)	Crystallite size^*a*^ (nm)	Surface area (m^2^ g^–1^)
SAS precipitation	100	18	32
Flame spray pyrolysis	90	40	15
Mechanochemical	95	25	1

^*a*^Determined from XRD analysis.

**Fig. 13:**
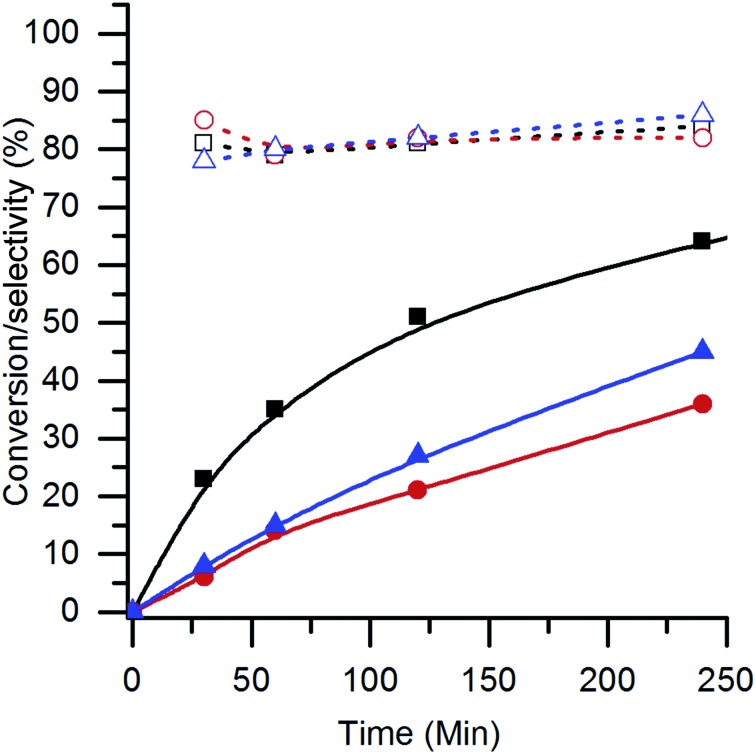
Conversion and C_3_ oxidation product selectivity of AuPt/LaMnO_3_ using different perovskite preparation routes. Conversions are represented with solid fills (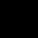 = SAS, 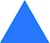 = flame pyrolysis and 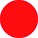 = mechanochemical) and selectivities with the corresponding open symbols.

After the addition of the 1 wt% AuPt, the LaMnO_3_ catalysts prepared by mechanochemical synthesis and flame pyrolysis were tested under the same glycerol oxidation reaction conditions as those used for the SAS catalysts (Fig. 13). It was evident that the conversion with these alternative supports was lower than that with the SAS prepared material and corresponded to the surface area of the supports. The reduced surface area would result in fewer sites for the AuPt nanoparticles, reducing the dispersion of the metals and, hence, lowering the conversions. Despite the reduced activity, a key observation was that all the catalysts tested had the same selectivity towards the C_3_ oxidation products (glyceric and tartronic acid). From an applied perspective, this is a key finding as it illustrates that the phenomenon of LaMnO_3_ supports inhibiting lactic acid production is not unique to any specific preparation technique. Provided a LaMnO_3_ support with sufficient surface area can be produced, a catalyst with strong selective oxidation potential can be synthesised.

## Conclusion

The SAS precipitation methodology has been successfully utilised as a method of catalyst discovery. More specifically, it has allowed access to a class of materials, namely perovskites, by providing sufficient surface areas to allow them to be used as supports for liquid phase glycerol oxidation. A range of LaBO_3_ supports (where B is Cr, Mn, Fe, Co or Ni) was prepared with surface areas between 22 and 52 m^2^ g^–1^. This allowed for the successful addition of 1 wt% AuPt nanoparticles to make active glycerol oxidation catalysts. Under the catalytic reaction conditions chosen, the selectivity of the reaction could be tuned between the production of glyceric/tartronic acid, through a sequential oxidation pathway, and the production of lactic acid, through an initial oxidation and dehydration pathway. The factor changing the reaction pathway was the choice of element in the perovskite B site, with Cr and Fe resulting in a high selectivity towards lactic acid, Mn being highly selective towards sequential oxidation products, and Co or Ni giving a range of products. The use of the single metal oxides as supports resulted in a loss of control of the product selectivity. Without the AuPt nanoparticles, the perovskite supports were inactive for the reaction. The choice of B site was found to have a negligible effect on the AuPt nanoparticle size but it did significantly alter the Au : Pt surface ratio, although there is little precedence in the literature for either Au or Pt having significantly different selectivities towards this reaction. A strong correlation was observed between the reported oxygen adsorption capacities of the different B site perovskites and the glycerol oxidation selectivity profile. A high oxygen adsorption capacity was found to correlate with the oxidation pathway and poor oxygen adsorption capacity for lactic acid formation.

Longer reaction times with the AuPt/LaMnO_3_ catalyst were found to give an exceptionally high tartronic acid yield of 88% at 100% glycerol conversion. Tartronic acid appeared to be predominantly formed from the sequential oxidation of glyceric acid. The comparable selectivity profile of AuPt/LaMnO_3_ prepared by flame pyrolysis and mechanochemical methods demonstrates the general applicability of perovskites as supports for selective oxidation reactions.
